# A topological analysis of targeted In-111 uptake in SPECT images of murine tumors

**DOI:** 10.1007/s00285-017-1184-8

**Published:** 2017-10-05

**Authors:** David B. Damiano, Melissa R. McGuirl

**Affiliations:** 1 Department of Mathematics and Computer Science, College of the Holy Cross, Worcester, MA USA; 2 Division of Applied Mathematics, Brown University, Providence, RI USA

**Keywords:** Computational topology, Persistent homology, Mathematical biology, Antibody uptake, Drug targeting, 92B05, 92C50, 92C55

## Abstract

Single-photon emission computed tomography images of murine tumors are interpreted as the values of functions on a three-dimensional domain. Motivated by Morse theory, the local maxima of the tumor image functions are analyzed. This analysis captures tumor heterogeneity that cannot be identified with standard measures. Utilizing decreasing sequences of uptake values to filter the images, a modified form of the standard persistence diagrams for 0-dimensional persistent homology as well as novel childhood diagrams are constructed. Applying statistical methods to time series of persistence and childhood diagrams detects heterogeneous uptake of radioactive antibody within tumors over time and distinguishes uptake in two groups of mice injected with different labeled antibodies.

## Introduction

Computational topology techniques have been utilized in image analysis, data analysis, bio-medicine, and other areas. They have been successfully applied to classify liver lesions in Adcock et al. (2012), to analyze brain artery trees in Bendich et al. (2016), and to measure shape of complicated geometric structures such as branched polymers in MacPherson and Schweinhart (2012). Here, we use persistent homology to analyze rates of antibody uptake and binding in a preclinical cancer study.

Since anti-cancer drugs are highly toxic, an important goal in drug design is to increase the amount of drug delivered to tumor while minimizing the amount delivered to healthy tissue and organs. Antibodies that bind to cancer specific antigens are commonly used as a vehicle for drug delivery. These antibodies are specific to the type of cancer and to the anti-cancer drug, and when effective are considered the “gold standard” of drug delivery (Maeda 2001). The labeling of antibodies with a radioactive isotope enables the study of antibody pharmacokinetics through various imaging modalities (Hoppin et al. 2010; Ma et al. 2014). Utilizing computational topology techniques, we have developed a method that is capable of quantifying local indium-111 (In-111) labeled antibody uptake behavior in a time series of single-photon emission computed tomography (SPECT) images of murine tumors.

An important consideration in the delivery of therapy to a tumor is the vascular structure of tumor tissue. Tumors promote the rapid extension of existing blood vessels into tumor tissue as well as the development of new blood vessels in the tumor. The resulting vessels are disorganized, have faulty architecture, and are unevenly distributed across the tumor. Often the well-vascularized tissue is situated along the periphery of the tumor, leaving a poorly vascularized hypoxic region that may include a necrotic core. Clinical testing has shown that up to 50–60% of locally advanced solid tumors have heterogeneously distributed regions of significant hypoxia (Denison and Bae 2012). This causes the heterogeneous distribution of therapy so that a large fraction of viable tumor cells may not be exposed to lethal doses of therapy (Tredan et al. 2007). Furthermore, this can promote drug resistance in localized regions of the tumor. In some cancers the presence of hypoxic regions correlates with a worse prognosis (Denison and Bae 2012). Consequently, it is important to develop methods for analyzing the heterogeneous distribution of therapy within a tumor.

Since antibodies are only delivered via blood, the heterogeneity in tumor vascular structure results in the heterogeneous distribution of antibody in the tumor. The standard measures of uptake of radiolabeled antibody are tumor average values normalized either by the average uptake of the tumor at an initial time or by the average value for healthy tissue. These are generally not able to capture heterogeneity of uptake. While there has been some work in this direction, prominent methods include working with a homogeneous sub-region of the image and reducing the tumor analysis to one radial direction, information is lost in this process (Yang and Knopp 2011).

We propose two new methods for detecting heterogeneity of uptake that utilize topological techniques and statistical methods to quantify antibody uptake over time. We have applied these methods to decay corrected and normalized SPECT images of murine tumors from a proprietary study which concerns the uptake behavior of a targeting antibody in comparison to a control antibody. Our methods differentiate between targeting and control antibody behavior as well as detect heterogeneity of uptake within tumors.

In Sect. 2 we provide a brief summary of the relevant biology and topology background pertaining to our new methods of analysis. An outline of our approach is presented in Sect. 3, where we introduce criteria for comparing the different methods. The results of our analysis are presented in Sect. 4. Specifically, in Sect. 4.1 we consider the effectiveness of standard methods of analysis, and in Sects. 4.2 and 4.3 we compare the results of the standard methods to different combinations of local methods (persistence diagrams and childhood diagrams), normalization, and data segmentation. In Sect. 5 we turn to possible biophysical explanations for these results.

## Background

### Tumor biology and drug targeting

Preclinical animal models of human cancers play an indispensable role in the process of drug discovery and development for new cancer drugs (Ruggeri et al. 2014). Murine or human (xenograft) tumors are induced in rodent models in a number of ways. Here we are concerned with ectopic subcutaneous tumors located on the hip of the mouse model. Cancers, including those in animal models, exhibit considerable intra-tumor heterogeneity (as well as inter-tumor heterogeneity) (Ruggeri et al. 2014). One cause of this heterogeneity is the random or haphazard structure of tumor vascular networks that results in complex branching patterns rather than the hierarchical branching of vasculature in normal tissue. Often the periphery of a tumor is well-vascularized whereas the interior is avascular (Yang and Knopp 2011). The formation of tumor vascular structure is due to angiogenesis, the extension of existing vessels, and vasculogenesis, the recruitment of circulating endothelial progenitor cells (Narang and Varia 2011).

**Fig. 1:**
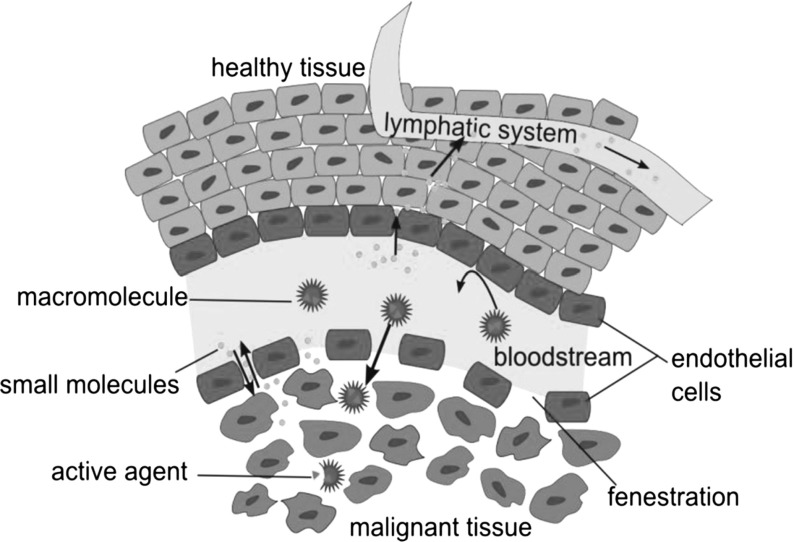
Schematic diagram of the enhanced permeation and retention effect on macromolecular extravasation in tumor tissue. Figure courtesy of Pharmaceuticals (Stockhofe et al. 2014)

The normal immune response includes factors that enhance the permeability of vessels, allowing for the accumulation of fluid and proteins in tissue from blood that aid in the immune response and account for localized swelling and pain. Angiogenic tumor vessels produce large amounts of vascular mediators that enhance their permeability (Maeda 2001). In addition, tumor vessels have defective endothelial cells with wide fenestrations and lack a muscle layer, which also promote permeability (Kobayashi et al. 2014). Moreover, the lymphatic clearance from the interstitial space of tumor tissue is impaired. The combined effects of the increased permeability of tumor vessels and the reduced clearance from tumor tissues is known as the *enhanced permeability and retention* (EPR) effect (Greish 2010; Heneweer et al. 2010; Maeda 2001; Srinivasan and Mane 2014). Figure 1 illustrates the EPR effect on macromolecular extravasation in tumor tissue.

An important therapeutic consequence of the EPR effect is that biocompatible macromolecules, including macromolecular drugs, accumulate at much higher concentrations in tumor tissue than in normal tissue or organs, even higher than those in plasma. This can be observed in macromolecules larger than 50 kDa, which have long plasma half-lives, since it takes at least 6 h for drugs in circulation to exert the EPR effect (Maeda 2001). Thus the EPR effect provides a passive delivery system for macromolecular or nano pharmaceuticals.

Active targeting of the tumor is accomplished by the use of cell-specific targeting ligands that can bind to surface receptors on tumor cells (Bartlett et al. 2007). One method of drug targeting involves attaching a toxic molecule to antibodies that bind to specific antigens on the surface of tumor cells. If the antibody successfully reaches the targeted tumor cells and binds to the targeted antigen, the antibody and toxin will be internalized by the cell allowing the toxin to kill the cell (Hoppin et al. 2010; Ma et al. 2014). Targeted cancer treatments have the potential to increase the amount of drug delivered directly to the tumor while minimizing the amount of treatment concentrated in healthy areas of the body.

When labeled with a radiopharmaceutical, various imaging modalities have been used to measure the biodistribution of nano molecules in tumor and healthy tissue (Hoppin et al. 2010). In this paper, we study CT-SPECT images of mice injected with targeting and non-targeting antibodies labeled with In-111. Standard measures for the analysis of these images include the percent injected dose per gram (%ID/g) of the tumor or region of interest (ROI) in an image, and the ratio of the %ID/g of the tumor to the %ID/g of healthy tissue. The quantity %ID/g is a measure of the uptake of labeled antibody per unit mass of tissue and is calculated for tumor or organ. It is the percentage of the total injected dose of labeled antibody observed in the tumor or organ per unit mass of the tumor or organ. It can be calculated by

1 $$\begin{aligned} \%\text {ID/g}= & {} \frac{\text{ uptake }}{\text{ injected } \text{ dose }} \times 100 \Big /\left( \frac{\text{ volume }}{1000}\right) , \end{aligned}$$

where uptake is the total of the SPECT intensity values summed over the entire tumor ROI or organ measured in micro-curies ($\mu$Ci), injected dose is the decay corrected injected dose of the probe (In-111 labeled antibody), and volume is the total volume of the ROI or organ in $\text{ mm }^3$. Tissue is assumed to have the density of water, so that the density of water can be used to convert volume to mass.

In this paper, normal tissue is represented by the heart, and thus the tumor-to-normal ratio is the ratio of the tumor ROI %ID/g to the heart %ID/g, which we denote by T:H. The tumor-to-normal ratio is computed for each image, normalizing uptake relative to that of healthy tissue. It can be used to detect the effects of the clearance of antibody in blood. This is of importance because the clearance rate of antibody in blood can affect the accumulation of antibody in tumor. If an antibody is cleared from the blood at a slow rate then it can result in higher tumor uptake compared to an antibody that clears the blood faster, masking the effect of antibody binding (Hoppin et al. 2010).

The decay of the radiopharmaceutical, which is independent of the binding of the antibody to tumor, decreases the amount of uptake behavior that can be captured through imaging (Hoppin et al. 2010). This is taken into account by decay correcting image data prior to the calculation of %ID/g. If $k = \ln (2)/\tau _{1/2}$ is the relative rate of decay of the radiopharmaceutical with half-life $\tau _{1/2}$, the data for an image *t* hours after the injection of the radiopharmaceutical is scaled by the factor $e^{kt}$.

Both %ID/g and the T:H ratio are aggregate measures obtained by averaging over the ROI. Consequently, they cannot be used to detect heterogeneity of uptake across the ROI. In an attempt to improve upon standard measures we apply topological techniques to analyze the local uptake of antibody in an ROI over time. These techniques will distinguish both inter- and intra-tumor heterogeneity that is not detectable by standard aggregate measures.

### Study data set

In this paper, we analyze a study data set consisting of 32 CT-SPECT (X-ray computed tomography and single photon emission computed tomography) images of 8 mice. Each animal in this study was injected with a non-xenograft tumor from the same mouse tumor cell line.

After the tumors reached approximately 400 mm$^3$, four of the mice were injected with In-111 labeled targeting antibody and the remaining four were injected with In-111 labeled non-targeting (control) antibody. Group 1 will refer to the four subjects injected with In-111 labeled targeting antibody (subjects T1–T4) and Group 2 will refer to the four subjects injected with In-111 labeled control antibody (subjects C1–C4). We will use this numbering throughout the paper. The targeting and control antibodies were the same size (150 kDa), and no treatment was given to any of the subjects in this study. The subjects in this trial were imaged at 3, 24, 48, and 72 h after injection, thus the total of 32 images. The image data have been decay corrected using $\tau _{1/2}=67.32$ h for the radioactive half-life of In-111.

**Fig. 2:**
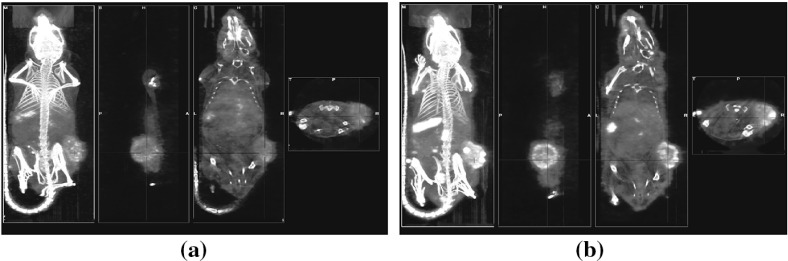
The CT image and sagittal, coronal, and transverse slices of SPECT images for subject T1 imaged at **a** 24 h and **b** 72 h. Images are exported from VivoQuant (Release 1.22, Invicro)

The CT image and sagittal, coronal, and transverse slices of two SPECT images for subject T1 are presented in Fig. 2. This figure shows the subject imaged 24 and 72 h after injection with the In-111 radiolabeled targeting antibody. The tumor is located on the right hip of the animal. In these images the bright regions correspond to areas of higher radioactivity. Note, the tumor as well as the heart and lungs show signs of high antibody uptake.

### Persistent homology and the structure of intensity functions

In Morse theory, the structure of a differentiable manifold is revealed through an analysis of the critical points, level sets, and superlevel (or sublevel) sets of smooth real-valued functions on the manifold (Milnor 1963). Here we apply discrete methods inspired by Morse theory to study heterogeneity of antibody uptake as revealed by the intensity of SPECT images of murine tumors over time. The analysis utilizes methods of computational topology, specifically, persistent homology in dimension 0. In general, persistent homology relies upon a choice of an increasing filtration of the manifold. Because we are interested in heterogeneity of antibody uptake, it will be convenient to use an increasing filtration by superlevel sets that is defined by a decreasing sequence of intensity values. Consequently, neighborhoods of local maxima of uptake, which carry information about heterogeneity of uptake, will be connected components of the sets in the filtration. In other applications it is more common to use sublevel sets and an increasing sequence of values, in which case neighborhoods of maxima would be contained in the complements of the sets of the filtration.

The SPECT images consist of three dimensional arrays of intensity values produced by ordered subset iterative reconstruction from SPECT photon counts. This method produces noise proportional to counts so that features of the image remain detectable in regions of low counts. The image data were smoothed in MATLAB (R2015a, Mathworks) using a Gaussian convolution kernel of box size $3 \times 3 \times 3$ (the smallest possible) and standard deviation 0.69. After minimal smoothing, array entries were interpreted to be the values of a function *f* on a rectangular box *D* containing the ROI with non-zero values of *f* corresponding to the ROI. Empirically, randomness inherent in smoothed SPECT values ensures that the critical points of *f* will be isolated and have distinct critical values. An intensity value of a voxel was determined to be a local maximum of *f* if it was strictly greater than the values in the remaining voxels of a $3 \times 3 \times 3$ box centered on the given voxel.^1^ Smoothing eliminates local maxima that may be due to noise. We will write $I = f(X)$ for the smoothed intensity of *f* at the point *X*, where *X* is a triple of integers indexing a voxel in the image. Here we interpret the indices as the coordinates of a point in *D* at the center of the voxel.

For an intensity value $I'$ the superlevel set $F'$ of *f* corresponding to $I'$ is the collection of voxels in the image for which $f(X) \ge I'$. If $I_1> I_2> \cdots > I_n$ is a decreasing sequence of non-zero values in the range of *f* with $I_n$ chosen to be less than the smallest positive value of *f*, then the corresponding increasing sequence of superlevel sets forms a filtration $F_1 \subset F_2 \subset \cdots \subset F_n$ of the superlevel set $F_n$. The condition on $I_n$ ensures that $F_n$ is equal to the ROI of the image. In practice, $F_n$ will be connected. In Sect. 3, we will construct filtrations so that for all *j*, $I_j$ is not a critical value of the intensity function and between $I_j$ and $I_{j+1}$ there is at most one critical value.

**Fig. 3:**
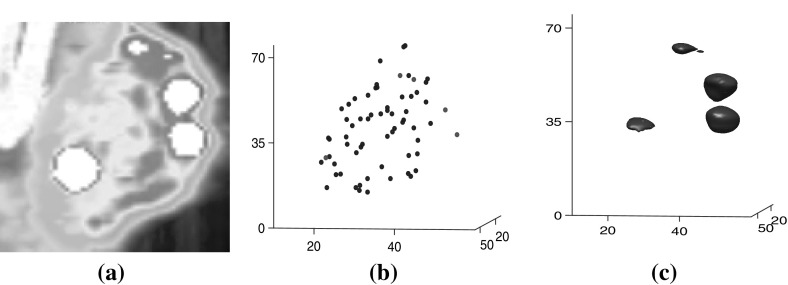
The T1 Hour 72 **a** cropped tumor image exported from VivoQuant (Release 1.22, Invicro), **b** local maxima with highest maxima in red, and **c** superlevel set neighborhoods of the five highest maxima for the same intensity value

Figure 3 contains a slice of the cropped SPECT tumor image for subject T1 at hour 72 (a), the local maxima of uptake of this image (b), and a superlevel set of the image (c). In Fig. 3c the superlevel set corresponds to an intensity value just below the value of the fifth highest maximum, and the superlevel set is a collection of neighborhoods of maxima, or connected components, surrounding the five highest maxima. Note that in Fig. 3a the horizontal axes have been flipped from Fig. 2b to match those of MATLAB (R2015a, Mathworks) images.

We are interested in the dimension zero persistent homology of filtrations of this form. Since the connected components of the sets $F_j$ can be identified with generators of the dimension zero homology of $F_j$, we will define the relevant concepts in terms of connected components rather than homology, thus avoiding a certain amount of notation.

A pair of voxels are considered to be adjacent if they share a vertex, edge, or face. A connected set of voxels is a set of voxels such that for any two voxels *X* and $X'$ in the set, there is a sequence $X = X_0, X_1, \ldots , X_l = X'$ of voxels in the set so that consecutive voxels in the sequence are adjacent.

Dimension zero persistent homology tracks the connected components of the $F_j$ as *j* increases. We will use the local maxima of uptake to label the connected components of $F_j$. Let $\{X_1, X_2, \ldots ,$ $X_m \}$ be the set of local maxima of uptake ordered so that $f(X_i) > f(X_{i+1})$. Each connected component *C* of $F_j$ contains one or more local maxima of *f*, so that we may think of the components of the $F_j$ as a collection of neighborhoods of the local maxima. If $X_{i_1}, X_{i_2}, \ldots , X_{i_k} \in C$, where $i_1> i_2> \cdots > i_k$, we will label *C* by $i_1$, the index of the local maximum in *C* with the largest critical value. We will write $C_{j,i}$ for the connected component of $F_j$ for which $X_i$ is the local maximum in the component with the largest value. Notice that not every *i* appears as an index of a connected component of $F_j$.

For each local maximum $X_i$, the collection of connected components $C_{j,i}$ forms a nested sequence $C_{j_b,i} \subset C_{j_b+1,i} \subset \cdots \subset C_{j_d-1,i}$ where $X_i$ is the maximum with largest value for each component in the sequence. Let $S_i$ denote the nested sequence corresponding to $X_i$. We will say that the nested sequence is *born at filtration value* $b_i = I_{j_b}$ and *dies at filtration value* $d_i=I_{j_d}$. Notice the death value occurs at the smallest filtration index $j_d$ such that $X_i \in C_{j_d,k}$ for some $k\ne i$. The largest maximum $X_k \in C_{j_d,k}$ necessarily satisfies $f(X_k) > f(X_i)$. Since filtration values decrease, the birth value of a subsequence is larger than the death value and the lifetime of a subsequence is the difference $b_i-d_i$.

Consider the component $C_{j_d,k}$ with $I_{j_d} = d_i$ that contains $X_i$. Because there is at most one critical value between every pair of filtration values, $C_{j_d-1,k}$ is the unique component of $F_{j_d-1}$ satisfying $C_{j_d-1,i} \cup C_{j_d-1,k} \subset C_{j_d,k}$. This is equivalent to saying that there is a single non-degenerate saddle with critical value between $I_{j_d -1}$ and $I_{j_d}$. The sequence $S_i$ is said to *join* or *merge* with the sequence $S_{k}$ at the filtration value $d_i$. It corresponds to a generator of the dimension 0 persistent homology of the filtration. We say that the generator *persists* from birth value $b_i$ to death value $d_i$. Our labeling convention for subsequences is equivalent to the *elder rule* in persistent homology (Edelsbrunner and Harer 2009).

Below we will have cause to use the initial subsequence of $S_i$ consisting of components which contain only the maximum $X_i$, $C_{j_b,i} \subset C_{j_b+1,i} \subset \cdots \subset C_{j_d'-1,i}.$ Since there is at least one filtration value between every pair of critical values, this subsequence is non-empty. Let $d'_i = I_{j'_d}$. There are two possibilities for $d'_i$. Either $d'_i = d_i$, so that $d'_i$ is the death value for the sequence, or $d'_i > d_i$ so that the sequence $S_k$ for a second, lower maximum $X_k$, $f(X_k) < f(X_i)$, joins the sequence $S_i$ for $X_i$ at filtration value $d_i'$ and the sequence $S_i$ persists beyond $d_i'$.

A persistence diagram is a scatter plot of points representing the subsequences $S_i$. Motivated by MacPherson and Schweinhart, we use a linear transformation of the birth and death values to create *persistence points* $(x_i,y_i) = (b_i - d_i,\frac{1}{2}(b_i + d_i))$ (MacPherson and Schweinhart 2012). We note that these coordinates are similar to the landscape coordinates of Bubenik (2015). We apply persistence diagrams, or persistent homology, to capture tumor heterogeneity through an analysis of neighborhoods surrounding local maxima throughout tumor ROIs. Persistence diagrams provide a method of quantifying the uptake behavior in these regions.

Typically, persistent homology has been applied to a filtration of the domain of a Morse function by an increasing sequence of sublevel sets, so that $b_i<d_i$. Most authors have represented connected components by the pair $(b_i,d_i)$ (Edelsbrunner and Harer 2009; Zomorodian 2009). With these conventions, information about connected components of maxima is captured by dimension 2 persistent homology. The two approaches can be shown to be equivalent by an application of Alexander duality.

### Childhood diagrams and binary trees

From a biological perspective, persistence points that represent components $C_i$ that die at the first opportunity are of particular interest. In terms of sequences of connected components of the filtration, these are sequences for which $d_i' = d_i$ and whose components only contain the maximum $X_i$. To use a geographic analogy, for these subsequences, the persistence coordinate $x_i=b_i-d_i$ is the height of the maximum $X_i$ above its base and the persistence coordinate $y_i = \frac{1}{2}(b_i + d_i)$ is the midway point of the climb from the base to the maximum. Thus $x_i$ and $y_i$ capture local information about the local maximum $X_i$. This is not the case for sequences that do not die at the first opportunity since they contain more than one local maximum.

At the extreme, the component $C_{n,1}$ that corresponds to the absolute maximum $X_1$ of intensity of uptake consists of the entire ROI, since $I_n$ is less than the minimum intensity value in the ROI and the ROI is connected. In order to recover local information about all local maxima, we introduce the concept of a *childhood diagram*. While this local uptake data will carry biological information, it does not carry topological information.

The childhood diagram of the filtration of an image is the collection of points $(x_i',y_i') = (b_i - d_i',\frac{1}{2}(b_i + d_i'))$. Thus for every local maximum, $x_i$ represents the difference between the uptake value at the maximum and the nearby background uptake and $y_i$ represents the average uptake value in the local neighborhood of the maximum. To understand the difference between the persistence diagram and the childhood diagram of an image it will be useful to introduce the binary tree for the image (Harris and Ross 2005). Binary trees of an increasing filtration of the domain of a Morse function are discussed in Edelsbrunner and Harer (2009), where they are called *merge trees*. Our use of binary trees is an example of a dendrogram in which the clusters are born at different filtration values [Statistics and Machine Learning Toolbox, MATLAB (R2015a, Mathworks)].

The binary tree of an image has *m* leaves or external branches $B_i$, $i=1,\ldots ,m$, and $m-1$ internal branches $B_i$, $i=m+1,\ldots , 2m-1$. The leaves and internal branches correspond to subsets of the set of maxima of the image and to subsequences of the sequences $S_i$ defined above. The leaves correspond to individual local maxima $X_i$ and to sequences $C_{j_b,i} \subset C_{j_b+1,i} \subset \cdots \subset C_{j_d'-1,i}.$ When two branches join at a node, the resulting internal branch is called the *parent* of the two joining branches and the joining branches are called the *children* of the parent. The set of maxima corresponding to a parent is the union of the sets of maxima corresponding to its children, and the unions of the connected components for the subsequences of children form disjoint neighborhoods in the connected components of their parent.

Each branch is assigned a birth value and a death value. For a leaf corresponding to the maximum $X_i$, the birth value $b_i$ is the value of the corresponding maximum and the death value $d_i'$ is as defined above. Leaves $B_i$ and $B_j$ that join at a node have a common death value, $d_i'=d_j'$. The birth value $b_i$ of an internal branch $B_i$ is the common death value of its children. The death value $d_i'$ of an internal branch is defined as above for maxima. It is the first value at which its set of maxima is augmented by one or more additional maxima resulting from joining with another branch.

**Fig. 4:**
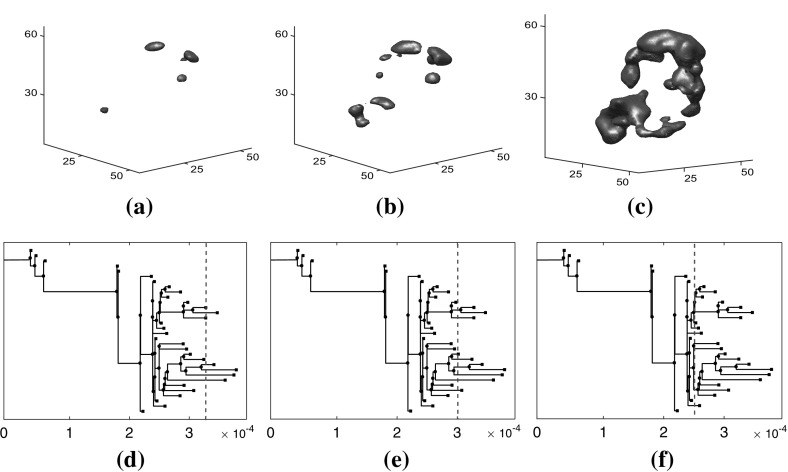
Superlevel sets of the SPECT image for T4 Hour 72 corresponding to decay corrected intensity values **a** 3.3722 $\times 10^{-04}$, **b** 3.0322$\times 10^{-04}$, and **c** $2.5021 \times 10^{-04}$. **d**–**f** Binary tree representations of the SPECT image for T4 Hour 72, where the portions of the tree to the right of the dashed line represent the connected components of the sequences $S_i$ for intensity values higher than the intensity values for subfigures (**a**)–(**c**)

Figure 4 demonstrates how binary trees can be used to represent the connected components of the superlevel sets of a SPECT image. Figure 4a–c show the superlevel sets of the SPECT image for T4 Hour 72, and Fig. 4d–f show the corresponding binary tree representations.

**Fig. 5:**
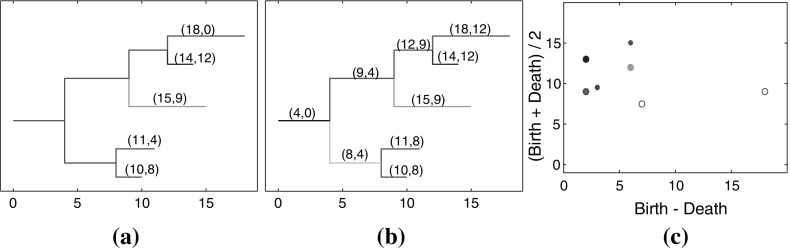
A binary tree color coded according to **a** persistence and **b** childhood, and **c** the corresponding superimposed persistence and childhood diagrams. Points are color coded by maximum, persistence points are filled-in and childhood points are hollow. The labels in (**a**) are the birth and death coordinates (*b*, *d*) for the leaf or branch and the labels in (**b**) are the birth and death coordinates $(b,d')$ for the leaf or branch

Figure 5 contains a binary tree colored and labeled to indicate the difference between persistence and childhood death values. In Fig. 5a the coloring of the leaves and internal branches is determined by the elder rule. At each node, the leaf with the larger birth value, that is, earlier in terms of the filtration index, persists. Each leaf is labeled with its birth value $b_i$ and death value $d_i$. For example, the leaf with the largest birth value $b_1=18$ necessarily persists to the root so that $d_1=0$. In Fig. 5b the leaves and internal branches have distinct colors, since the elder rule does not apply. They are labeled with their birth value $b_i$ and death value $d_i'$, where $d_i'$ is the value at which the leaf or internal branch joins with its sibling. For example, the leaf with the largest birth value has death value $d_1'=12$. The scatter plot in Fig. 5c contains the persistence points and childhood points for this tree.

**Fig. 6:**
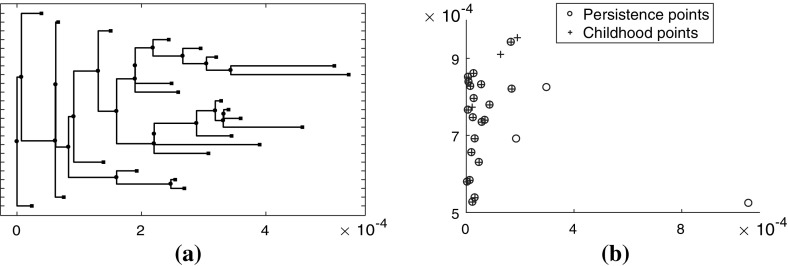
**a** Binary tree representation of image T4 H24, and **b** normalized persistence points and childhood points corresponding to image T4 H24 [normalization accounts for the discrepancy between the scales (**a**) and (**b**)]

Figure 6a shows the tree for subject T4 imaged at 24 h and Fig. 6b shows the corresponding persistence and childhood diagrams on the same axes. We can see that the points for the largest leaves move up and to the left in the childhood diagram compared to the corresponding persistence points. Note that in Fig. 6a the resolution of the image is not sufficient to capture the small differences in death values of certain leaves and branches. Consequently, the tree may not appear binary in places where adjacent leaves and branches have close death values.

In the persistence diagram the death value of the highest maximum is zero and therefore $(b-d, \frac{b + d}{2}) = (b, \frac{b}{2})$. This holds for the highest maximum across all images, and thus persistence diagrams fail to capture distinguishing, local features from regions with the most antibody uptake. The effect of switching to childhood lifetimes is to increase the death values of the high persistence points while not significantly changing the death values of the low persistence points. In moving from persistence diagrams to childhood diagrams the high persistence points move up and to the left, revealing local information from high uptake regions.

The first childhood coordinate $b-d$ is a measure of the local heterogeneity of uptake. Small values reflect local maxima not far above the background uptake. Decreasing values of $b-d$ in a region of the ROI reflect decreasing heterogeneity in the region. The second childhood coordinate $\frac{b+d}{2}$ is a measure of the uptake in the local neighborhood of a maximum. These values may move independently over time. We note that in some applications persistence points with small $b{-}d$, which represent short-lived topological features with regard to the filtration, are not considered to be important and are not included in the analysis. Because of our interest in heterogeneity of uptake, we have retained all persistence and childhood points in the analyses, including those with small $b{-}d$.

There is another independent argument for focusing on childhood diagrams rather than persistence diagrams having to do with phylogenetic distances on the space of labeled trees. In an analysis (not presented here) of the distances between the trees for the images of this data set, we showed that the distinguishing information was contained in the lengths of the largest leaves of the trees.

In Sect. 4.3 we will show by a statistical analysis of time series of persistence diagrams and of childhood diagrams that childhood diagrams contain more information in the sense that the analysis of childhood diagrams is better able to detect heterogeneity of uptake and local uptake behavior than that of persistence diagrams. In Sects. 5.1 and 5.2 we discuss why time series of childhood diagrams surpass time series of persistence diagrams in their ability to differentiate uptake behavior of the targeting antibody from that of the control antibody and why persistence and childhood diagrams detect heterogeneity of uptake behavior that cannot be captured with %ID/g and T:H ratio.

## Approach

The approach we take for quantifying the binding rates of targeting antibodies and heterogeneity of uptake within the tumors employs statistical analyses of time series of persistence and childhood diagrams. The diagrams are of the zero dimensional persistent homology of the image filtered by intensity. This method was implemented in a MATLAB (R2015a, Mathworks) pipeline that takes as its input a time series of raw data and ROI images exported from VivoQuant (Release 1.22, Invicro), a post-processing image analysis software, and produces both a time series of persistence diagrams and a time series of childhood diagrams. Persistence diagrams and childhood diagrams contain information about the intensity behavior of SPECT images in neighborhoods of local maxima in tumor ROIs. Combining the persistence diagrams and the childhood diagrams of a series of images of an individual subject taken at subsequent times produces time series persistence diagrams and time series childhood diagrams for the subject. Combining the time series diagrams for the subjects in a group produces time series persistence diagrams and childhood diagrams for the different groups.

In order to allow for the distribution of antibody in tissue and for EPR to take effect, the hour 3 data were not included in the time series analyses. There are two phases of antibody clearance observed in blood. In the first phase, the antibody distributes from blood into tissue. The elimination of the antibody from the body occurs in the second phase. By discarding the hour 3 data, the analysis begins in the second phase of antibody clearance.

Data were decay corrected and smoothed (see Sect. 2.3) before being normalized. Two normalization methods were applied. Data for each subject were normalized by the mean intensity value of uptake for the hour 24 ROI for the subject (H24 normalization). Separately, data for each subject at each hour were normalized by the mean intensity value of the heart uptake at that hour (heart normalization). H24 normalization makes it possible to compare the rates of change of uptake for different subjects from a common baseline value, thus facilitating intra- as well as inter-group comparisons. Heart normalization eliminates the effects of the removal of antibody from blood by natural processes, making it possible to observe changes in binding over time for an individual subject. This facilitates intra- and inter-group comparisons of binding rates over time. The analysis of the persistence diagrams and childhood diagrams normalized by the hour 24 ROI mean value was compared to the standard %ID/g calculations normalized by the hour 24 %ID/g value for the subject. Analogously, the analysis of the persistence diagrams and childhood diagrams normalized by mean heart uptake was compared to the standard T:H calculations.

In order to analyze uptake behavior in different regions of the tumor ROI, the persistence and childhood points were separated into high and low subsets based on the value of their corresponding maxima. The high and low subsets were determined on an image by image basis before aggregating across times and subjects within a group. The high persistence and childhood points of an image consist of the points corresponding to maxima whose values are at or above the 95th percentile of maximum values for the image. The low persistence and childhood points consist of points corresponding to maxima below the 95th percentile of maximum values for the image. We also carried out separate analyses using the 80th and 85th percentiles. The results (not shown) were similar to those for the 95th percentile. However, since the phylogenetic analysis described above indicated the importance of the small number of leaves corresponding to the highest maxima, we have chosen to report only the results for the 95th percentile-based analysis, which captures only the highest maxima. The analyses of time series of high and low persistence and childhood points were compared to analyses of H24 normalized %ID/g data and T:H data on regions of high uptake and of low uptake in the ROI, where the 95th percentiles of the non-zero values in the tumor ROIs were used as the threshold for separating high and low regions of the ROI.

Taking a step back, we have two standard aggregate methods for analyzing uptake, H24 normalization of %ID/g and heart normalization of %ID/g, and four novel methods for analyzing uptake, H24 normalized and heart normalized persistence diagrams and H24 normalized and heart normalized childhood diagrams. Statistical tests are applied to both the *x*- and *y*-coordinates of these diagrams. Further, each of these methods can be applied to segmented data. The segmentation for the standard methods is determined by the 95th percentile of intensity values in the ROI of an image. The segmentation for the persistence and childhood methods is based upon the 95th percentile of the values of the local maxima in the image.

The large number of methods combined with the possibilities for segmentation presents two challenges: what criteria should be used to assess the effectiveness of a combination and how can they be used to determine which combination is more advantageous in detecting differences in uptake between the two study groups and in detecting heterogeneity of uptake within a group.

To evaluate the different methods of analysis, we focus on the following three properties that a method might satisfy:

(i) it directly distinguishes between targeting and control antibody uptake rates,
(ii) it directly distinguishes between in-group high and low region uptake rates,
(iii) it distinguishes between targeting and control antibody uptake indirectly by displaying different patterns of statistical significance in the in-group high and low region uptake rates.

The first property concerns between group comparisons, while the second and third concern within-group comparisons. Notice that the third property can only be satisfied if the second property is satisfied for one group but not the other. Thus, the first property addresses the method’s ability to capture the effectiveness of the targeting antibody, whereas the second and third address the method’s ability to capture tumor heterogeneity.

Because there are several factors acting in conjunction to affect the uptake of antibody by tumor, including the binding rate of antibody to antigen, the EPR effect, the clearance rate of antibody from blood, and the decay of the radioactive tracer, it is too much to expect any one method to disentangle the effects of the different factors. However, applying two or more methods may begin to distinguish the different biological effects. Minimally, for a method to be effective in a particular context, it should be able to distinguish the behavior of the uptake of the targeting and control antibodies, that is, satisfy the first property.

Here, we are interested in detecting and understanding heterogeneous uptake behavior within groups and using this behavior to distinguish groups. Hence, we are most interested in identifying methods that satisfy the second and third properties.

## Results

In the following sections we present normalized results of standard methods of analysis, time series persistence diagrams, and time series childhood diagrams, as well as corresponding statistical analyses of these methods. To determine the usefulness of the standard methods, persistence points, and childhood points we will refer to the three criteria presented in Sect. 3.

For the purpose of this analysis, when applying trend comparison analysis to the different combinations of method, normalization, and data segmentation, all trends were assumed to be linear. In the statistical analyses, *p* values greater than 0.05 will be reported as not significant, *p* values with $0.01< p < 0.05$ will be reported as significant, and *p* values less than 0.01 will be reported as highly significant. See the Appendix for exact *p* values, the slopes of the best fit lines of the data, and corresponding figures.

### Standard analyses: H24 normalized %ID/g and T:H ratio

The standard measures used for comparison of antibody uptake in tumor to healthy tissue and organs are %ID/g and T:H ratio. Data were decay corrected prior to these calculations. %ID/g and T:H ratio were calculated on the entire tumor, as well as separately for high and low regions of the tumor. High regions consist of voxels whose value is in the 95th percentile of the tumor voxel values and low regions consist of voxels whose value is less than the 95th percentile of tumor voxel values.

The calculations show that Group 1 H24 normalized %ID/g consistently decreases over time, whereas Group 2 H24 normalized %ID/g is not consistent across all subjects (see Fig.  8). In contrast, the T:H ratio values increase over time in both Group 1 and Group 2 (see Fig. 9). We note that in both Group 1 and Group 2 the heart %ID/g values decrease over time, while the rate of decrease is about 25% greater for the Group 1 heart %ID/g than for the Group 2 heart %ID/g.

Linear trend comparisons show that the difference between the slopes of the Group 1 and Group 2 H24 normalized %ID/g calculations is significant, while the difference between the slopes of the Group 1 and Group 2 T:H ratio calculations is not significant (see Table 3a and Fig. 10). When the H24 normalized %ID/g calculations are separately repeated on high and low regions of the tumor, %ID/g is able to distinguish uptake behavior in regions of high uptake in Group 1 from the uptake behavior in regions of high uptake in Group 2. In contrast, %ID/g fails to distinguish uptake behavior in regions of low uptake in Group 1 from uptake behavior in regions of low uptake in of Group 2 (see Table 3a). Moreover, H24 normalized %ID/g fails to distinguish uptake rates between regions of high and low uptake within both groups (see Table 4a). Thus, H24 normalized %ID/g has property (i) in regions of high antibody concentration and over the whole tumor ROI, while %ID/g does not have property (i) in regions of low antibody concentration, property (ii) in either Group 1 or Group 2, or property (iii).

Linear fit comparisons of the T:H ratio calculations show that T:H ratio fails to directly distinguish between targeting and control uptake rates in all, high, and low regions of antibody concentration (see Table 3a). In contrast to H24 normalized %ID/g, the differences between the T:H uptake values in regions of high uptake and the T:H uptake values in regions of low uptake are highly significant in both Group 1 and Group 2 (see Table 4a). Thus, T:H ratio has property (ii) in both Group 1 and Group 2, but T:H ratio does not have property (i) in all, high, or low regions of the tumor or property (iii).

### Persistence diagrams and childhood diagrams

**Fig. 7:**
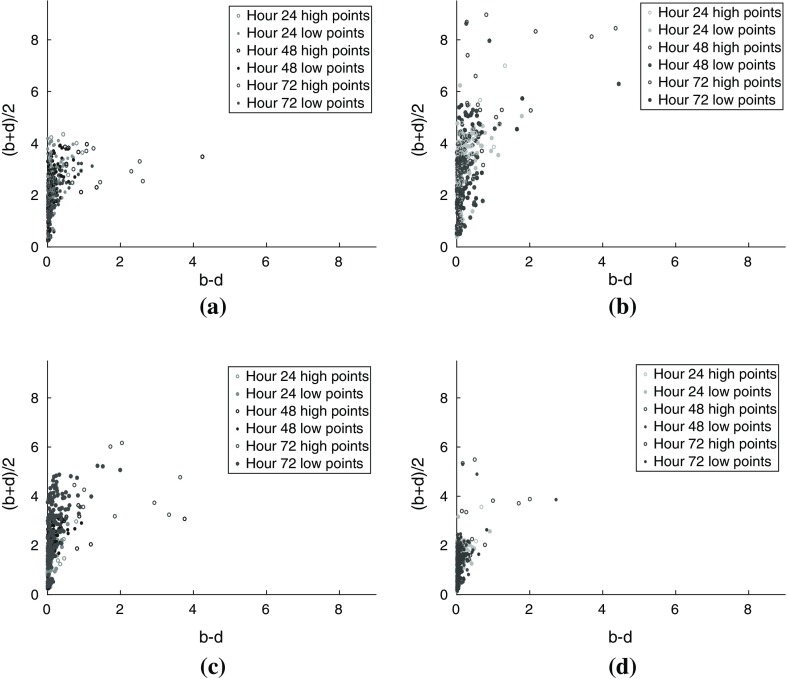
Time series childhood diagrams corresponding to **a** Group 1 H24 normalized data, **b** Group 1 heart normalized data, **c** Group 2 H24 normalized data, and **d** Group 2 heart normalized data

**Fig. 8:**
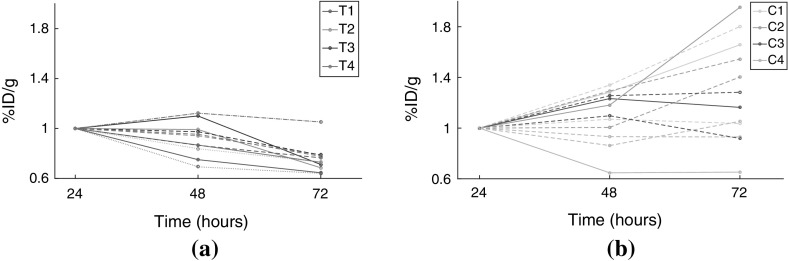
Tumor ROI H24 normalized %ID/g calculations for **a** Group 1 and **b** Group 2. The solid lines correspond to the calculations computed on all voxels, the dashed lines correspond to calculations computed on high voxels, and the dotted lines correspond to calculations computed on low voxels. Group 1 H24 normalized %ID/g values consistently decrease over time, whereas Group 2 H24 normalized %ID/g values are not consistent across all subjects

For each normalization of Group 1 and of Group 2, the persistence points of all subjects in the group were combined to create aggregate time series persistence diagrams. Similarly, for each normalization of Group 1 and of Group 2, the childhood points of all subjects in the group were combined to create aggregate time series childhood diagrams. These are presented in Fig. 7.

We are interested in analyzing the change in the positions of the childhood points over time both across and within groups. Figure 7a, b show the H24 normalized time series childhood diagrams for Groups 1 and 2 respectively, and Fig. 7c, d show the heart normalized time series childhood diagrams for Groups 1 and 2 respectively.

The *x*-coordinates do not appear to change over time in the H24 normalized diagrams in Fig. 7a, b, but they tend to increase over time in the heart normalized diagrams in Fig. 7c, d. In the H24 normalized time series diagrams, the Group 1 *y*-coordinates appear to decrease over time in Fig. 7a, whereas there is no apparent trend for the Group 2 *y*-coordinates in Fig. 7b. In contrast, in the Group 1 heart normalized time series diagram in Fig. 7c, the *y*-coordinates tend to increase over time, while there is no clear trend for the Group 2 *y*-coordinates in Fig. 7d. With the exception of the high persistence points, the change in the *x*- and *y*-coordinates over time is consistent across both the time series persistence diagrams and the time series childhood diagrams. We turn to statistical analyses to quantify these observations.

### Statistical analyses of persistence diagrams and childhood diagrams

Kruskal–Wallis testing and trend comparison analysis were applied to the time series of all persistence points and all childhood points, as well as to the separate high and low time series of persistence points and childhood points (Neter et al. 1990; Oehlert 2000). These tests were applied to aggregate time series persistence points and time series childhood points comparing all of the points in Group 1 against all of the points in Group 2.

For both the persistence and childhood points, the Kruskal–Wallis test indicates that the change over time of the H24 normalized *y*-coordinates, heart normalized *x*- and *y*-coordinates is significant in both Group 1 and Group 2 (see Table 1b and Figs. 11, 12). In contrast, Kruskal–Wallis testing showed that the change in the H24 normalized *x*-coordinates over time was not significant so these coordinates were not analyzed further. This indicates that for H24 normalization, the local heterogeneity of uptake as measured by $b{-}d$ did not change over the period of the study.

Based upon these results, trend comparison analysis was applied to the H24 normalized *y*-coordinates and heart normalized *x*- and *y*-coordinates of the persistence points and childhood points to distinguish the rate of change of uptake over time between Group 1 and Group 2, and to analyze heterogenous uptake behavior. Since the childhood coordinates surpass the persistence coordinates with respect to the three criteria presented in Sect. 3, we restrict our discussion in this section to the linear trend comparisons applied to the childhood coordinates here and direct the reader to the Appendix for the linear trend comparisons of the persistence coordinates (Tables 2, 3, 4).

We begin with the analysis of the H24 normalized *y*-coordinates. Linear trend comparisons demonstrate that the difference between the Group 1 and Group 2 slopes of the childhood *y*-coordinates is highly significant with respect to the H24 normalization. When separated into high and low groups, the difference between the slopes of the Group 1 and Group 2 high childhood *y*-coordinates is significant and the difference between the slopes of the Group 1 and Group 2 low childhood *y*-coordinates is highly significant (see Table 3b).

Within-group linear trend comparisons were applied to the high and low points to test for heterogeneous uptake behavior between segmented tumor regions with respect to the H24 normalization. The difference in slopes of the Group 1 high and Group 1 low H24 normalized childhood *y*-coordinates is not significant, while the difference in slopes of the Group 2 high and Group 2 low H24 normalized childhood *y*-coordinates is significant (see Table 4b and Fig.  13). Thus, the H24 normalized *y*-coordinates of the childhood points have property (i) in all, high, and low regions of the tumor, and these coordinates have property (iii) since they have property (ii) in Group 2 but not in Group 1.

We turn now to the analysis of the heart normalized *x*-coordinates. Linear trend comparisons show the difference between the Group 1 and Group 2 slopes of the childhood *x*-coordinates is highly significant with respect to the heart normalization. When separated into high and low groups, the differences between the slopes of the Group 1 and Group 2 high heart normalized childhood *x*-coordinates and between the slopes of the Group 1 and Group 2 low heart normalized childhood *x*-coordinates are highly significant (see Table 3b).

Linear trend comparisons were also applied to the high and low childhood points within each group. In both Group 1 and Group 2, the difference between the slopes of the high and low childhood *x*-coordinates, are highly significant (see Table 4b and Fig. 14). These results show that the heart normalized *x*-coordinates of the childhood points have property (i) in all, high, and low regions of the tumor, and these coordinates have property (ii) in both Group 1 and Group 2, but they do not have property (iii).

Lastly, trend comparison analysis applied to the heart normalized *y*-coordinates indicates that the difference between the slopes of the Group 1 and Group 2 heart normalized childhood *y*-coordinates is highly significant. When the childhood points are separated into high and low groups, the difference between the slopes of the Group 1 and Group 2 high heart normalized childhood *y*-coordinates is not statistically significant whereas the differences between the slopes of the Group 1 and Group 2 low heart normalized childhood *y*-coordinates are highly significant (see Table 3b).

The within-group linear trend comparisons show that the difference between the slopes of the Group 1 high and Group 1 low heart normalized childhood *y*-coordinates is significant, and the difference between the slopes of the Group 2 high and Group 2 low H24 normalized childhood *y*-coordinates is highly significant (see Table 4b and Fig. 15). These results show that the heart normalized *y*-coordinates of the childhood points have property (i) in the whole tumor ROI and in regions of low uptake, but not in regions of high uptake. This suggests that with respect to the heart normalized data the distinguishing features between uptake of the targeting antibody and uptake of the control antibody come from areas of lower uptake. As was the case with the heart normalized *x*-coordinates, trend comparison analysis applied to the heart normalized *y*-coordinates shows that the heart normalized *y*-coordinates of the childhood points have property (ii) in both Group 1 and Group 2 and thus they do not have property (iii).

To summarize, trend comparisons of the rates of change of the coordinates of childhood points demonstrate the ability of childhood diagrams to distinguish uptake of targeting antibody and control antibody and to identify heterogeneity of uptake. Using H24 normalization, *y*-coordinates of childhood points distinguish rates of uptake overall and in regions of high and low uptake. Within groups, *y*-coordinates of childhood points detect heterogeneity between high and low regions in Group 2 and homogeneous behavior in Group 1. Turning to the heart normalized data, the trend comparison analysis of childhood *x*-coordinates distinguishes overall uptake rates between groups and uptake in both high and low regions between groups. Further, the trend analysis detects heterogeneous uptake behavior between high and low regions in both groups when applied to the heart normalized childhood *x*-coordinates. Lastly, the heart normalized *y*-coordinates of the childhood points distinguish overall rates of uptake and rates of uptake in low regions between the two groups, and detects differences in uptake rates between high and low regions in both groups.

## Interpretation of results

### Between-group comparisons

The slopes of the H24 normalized %ID/g calculations and the slopes of the H24 normalized *y*-coordinates of both the persistence points and the childhood points are negative for Group 1 and positive for Group 2 (Table 2). Consequently, the H24 normalized %ID/g calculations and the H24 normalized *y*-coordinates of both persistence and childhood points successfully distinguish the clearance rates of the targeting antibody from that of the control antibody when looking at the whole tumor ROI (Table 3).

**Fig. 9:**
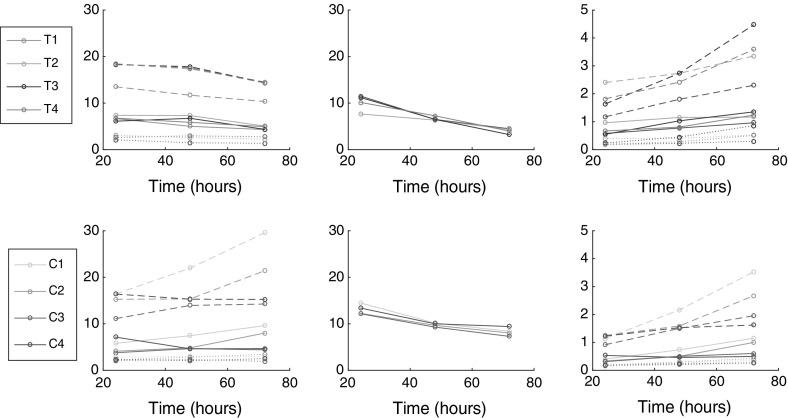
Tumor ROI %ID/g, heart ROI %ID/g, and T:H ratio calculations from left to right with Group 1 data in the top row and Group 2 data in the bottom row. The solid lines correspond to the calculations computed on all voxels, the dashed lines correspond to calculations computed on high voxels, and the dotted lines correspond to calculations computed on low voxels. The rate of decrease is about 25% greater for the Group 1 heart %ID/g than for the Group 2 heart %ID/g

**Fig. 10:**
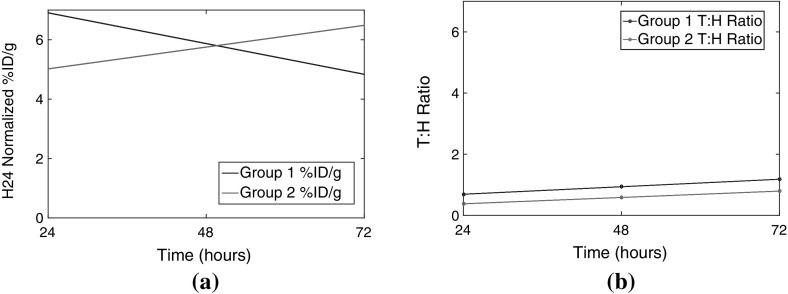
Best fit lines of the **a** H24 normalized %ID/g values and **b** T:H ratio values. Trend comparisons of Group 1 and Group 2 show the difference in slopes is significant for (**a**) and not significant for (**b**)

**Fig. 11:**
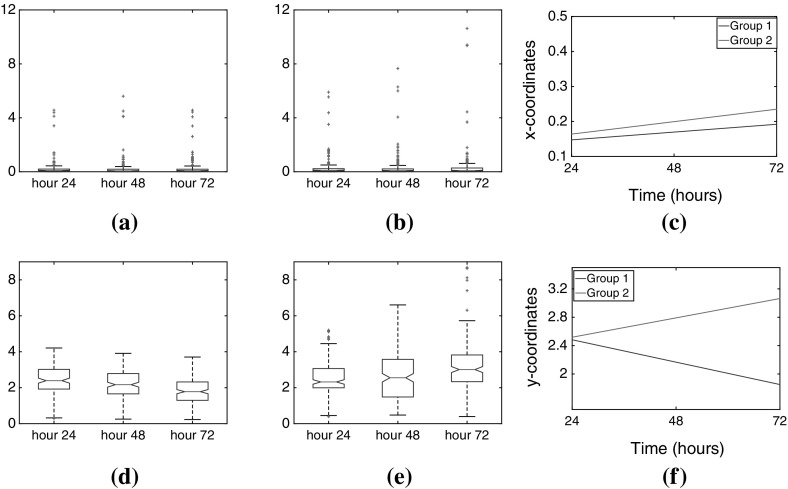
Notched box plots of the H24 normalized childhood points for the **a** Group 1 *x*-coordinates, **b** Group 2 *x*-coordinates, **d** Group 1 *y*-coordinates, and **e** Group 2 *y*-coordinates, and the best fit lines of the H24 normalized (**c**) *x*-coordinates and **f** *y*-coordinates of the childhood points. If the notches do not overlap then the distribution of the coordinates is significantly different across times at the 5% significance level. Kruskal–Wallis testing indicates that the change in distribution of *x*-coordinates is not significant across times in both Group 1 (**a**) and Group 2 (**b**), while the change in distribution of *y*-coordinates is highly significant across times in both Group 1 (**c**) and Group 2 (**d**)

**Fig. 12:**
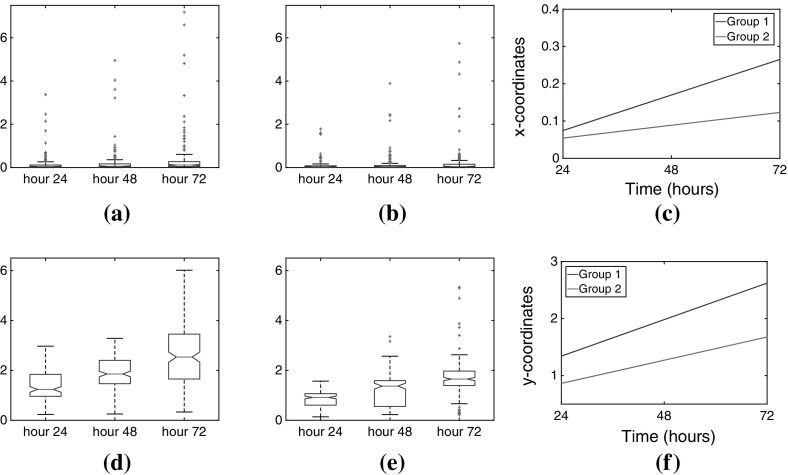
Notched box plots of the heart normalized childhood points for the (a) Group 1 *x*-coordinates, (b) Group 2 *x*-coordinates, (d) Group 1 *y*-coordinates, and (e) Group 2 *y*-coordinates, and the best fit lines of the heart normalized (c) *x*-coordinates and (f) *y*-coordinates of the childhood points. If the notches do not overlap then the distribution of the coordinates is significantly different across times at the 5% significance level. Kruskal–Wallis testing indicates that the change in distribution of *x*-coordinates is highly significant across times in both Group 1 (a) and Group 2 (b), and the change in distribution of *y*-coordinates is also highly significant across times in both Group 1 (d) and Group 2 (e)

**Fig. 13:**
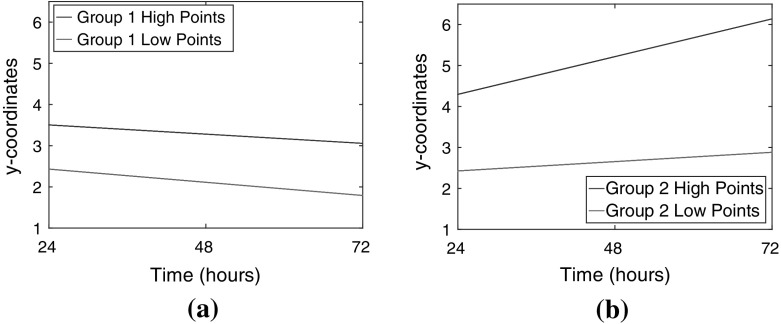
Best fit lines of the high and low H24 normalized *y*-coordinates of childhood points for **a** Group 1 and **b** Group 2. Trend comparisons of the high and low points show the difference in slopes is not significant for **a** Group 1, but is significant for **b** Group 2

**Fig. 14:**
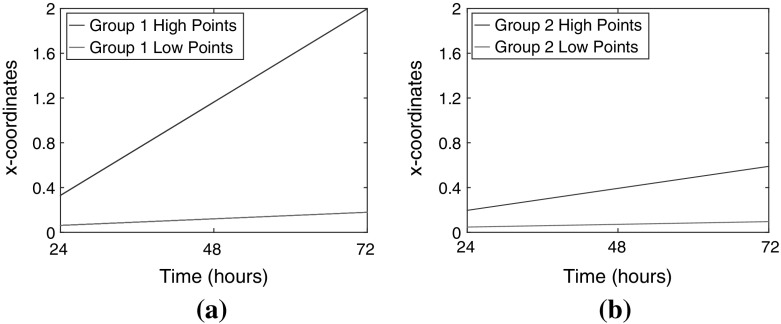
Best fit lines of the high and low heart normalized *x*-coordinates of childhood points for **a** Group 1 and **b** Group 2. Trend comparisons of the high and low points show the difference in slopes is highly significant for both **a** Group 1 and **b** Group 2

**Fig. 15:**
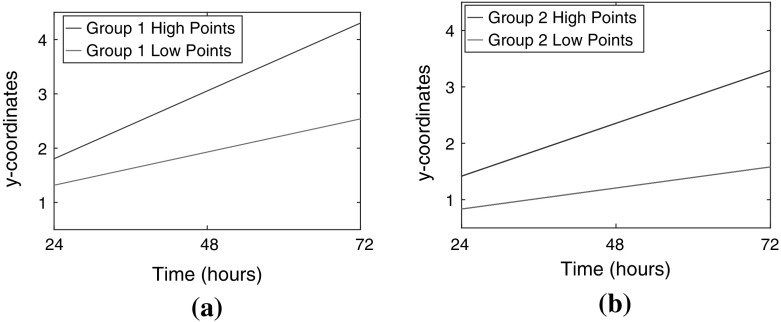
Best fit lines of the high and low heart normalized *y*-coordinates of childhood points for **a** Group 1 and **b** Group 2. Trend comparisons of the high and low points show the difference in slopes is significant for **a** Group 1 and highly significant for **b** Group 2

To understand the discrepancy in the sign of the slopes we consider the heart %ID/g calculations for both groups. For each group the heart %ID/g values decrease over time, while the rate of decrease is about 25% greater for the Group 1 heart %ID/g than for the Group 2 heart %ID/g (Fig. 9). These slopes are measures of the rate of antibody clearance in blood and so the results show that the targeting antibody clears at a faster rate than the control antibody.

The targeting antibody and control antibody are the same size (150 kDa), and therefore we expect the EPR effect to cause both the targeting and control antibody to accumulate in the tumor tissue at approximately the same rate. This buildup is opposed by the clearance of antibody from blood and, in the case of the targeting antibody, binding to tumor cell receptors, internalization by the tumor cells, and degradation by the cell (Greish 2010; Heneweer et al. 2010; Srinivasan and Mane 2014). These rates are unknown.

Combining these considerations, the decline in the Group 1 H24 normalized %ID/g and the H24 normalized *y*-coordinates of the persistence points and of the childhood points can be accounted for by a rate of clearance of the targeting antibody that is greater than the combined rate of buildup of targeting antibody in tumor that is due to the EPR effect and the rate of binding of the targeting antibody to tumor. In contrast, the increase in the Group 2 H24 normalized %ID/g and H24 normalized *y*-coordinates of the persistence points and of the childhood points can be accounted for by the rate of buildup of control antibody in tumor due to EPR being greater than the rate of clearance of the control antibody from blood. Consequently, the H24 normalized %ID/g calculations, the H24 normalized persistence points, and the H24 normalized childhood points can distinguish clearance and accumulation rates in Group 1 from clearance and accumulation rates in Group 2.

When the tumor ROI is segmented into regions of high and low uptake, H24 normalized %ID/g can distinguish Group 1 from Group 2 when comparing regions of high uptake but it fails to distinguish Group 1 from Group 2 when comparing regions of low uptake. In contrast, the H24 normalized *y* persistence coordinates are able to distinguish Group 1 from Group 2 in regions of low uptake but not in regions of high uptake, and the H24 normalized *y* childhood coordinates are able to distinguish Group 1 from Group 2 in regions of both high and low uptake (Table 3).

Since the EPR effect is greater in regions of high uptake, %ID/g cannot distinguish Group 1 from Group 2 in regions where the antibodies are accumulating at a slower rate (Greish 2010; Heneweer et al. 2010; Srinivasan and Mane 2014). While there is a greater antibody concentration in region of high uptake, regions of low uptake occupy a greater proportion of the tumor ROI. Moreover, since low death values inhibit the high persistence points’ ability to capture distinguishing local features, the H24 normalized *y*-coordinates of the persistence points cannot distinguish Group 1 from Group 2 in regions of high uptake. Thus, the H24 normalized *y* childhood coordinates can identify distinguishing features that cannot be identified with global measures. In summary, of the three H24 normalized methods, *y*-coordinates of childhood points are best able to capture distinguishing features of uptake behavior in local regions of the tumors.

T:H ratio and the heart normalized *x* persistence coordinates fail to distinguish the difference in uptake rates between Group 1 and Group 2, while the heart normalized *y* persistence coordinates and the heart normalized *x* and *y* childhood coordinates are able to distinguish Group 1 uptake rates from Group 2 uptake rates (Table 3). For both the *x*- and *y*-coordinates, the slopes of the heart normalized persistence points and of the heart normalized childhood points are greater in Group 1 than in Group 2, but the slopes are positive for both groups (Table 2b, c). The heart normalized y-coordinates show that the binding rates are greater with the targeting antibody than the control antibody, whereas the increase in heart normalized x-coordinates shows that there is an increase in local heterogeneity of uptake over time and this effect is greater in Group 1 than in Group 2. Since T:H ratio and the heart normalized *x* persistence coordinates measure uptake and heterogeneity on a global scale these measures cannot capture the binding effects of the targeting antibody.

When the tumor ROI is segmented into regions of high and low uptake, T:H ratio fails to distinguish Group 1 from Group 2 in the high and low regions. In contrast, the heart normalized *x*- and *y*-coordinates of the persistence points are able to distinguish Group 1 from Group 2 in regions of low uptake, but not in regions of high uptake. The heart normalized *x*-coordinates of the childhood points are able to distinguish Group 1 from Group 2 in high and low regions, while the heart normalized *y*-coordinates of the childhood points can differentiate Group 1 from Group 2 in regions of low uptake but not in regions of high uptake (Table 3).

The heart normalized *x*-coordinates of the childhood coordinates can distinguish Group 1 from Group 2 in both high and low regions of the tumor, and the slope of the Group 1 heart normalized *x*-coordinates is greater than the slope of the Group 2 heart normalized *x*-coordinates. This difference indicates that heterogeneity of antibody accumulation in the neighborhoods of maxima is more prominent with the targeting antibodies than the control antibodies after controlling for clearance. The contrast between the Group 1 and Group 2 heart normalized *x*-coordinates shows that the childhood points capture the heterogenous binding effects of the targeting antibody in regions of high and low uptake.

The heart normalized *y*-coordinates of the childhood coordinates fail to distinguish between the high uptake regions of Group 1 and the high uptake regions of Group 2. A possible explanation for this behavior is that in regions of high uptake the EPR effect masks the binding effects of the targeting antibody. It is more difficult to distinguish Group 1 and Group 2 in regions of high EPR since the EPR effect is primarily dependent on the size of the antibody, and the targeting and control antibodies are approximately the same size. Thus, of the four heart-normalized methods, *x*-coordinates of childhood points are best able to capture distinguishing features of uptake behavior between groups in segmented regions of the tumors.

With respect to both H24 normalization and heart normalization, childhood diagrams surpass both standard measures and persistence diagrams in their ability to differentiate uptake behavior of the targeting antibody from that of the control antibody. In particular, H24 normalized *y*-coordinates of childhood points and heart normalized *x*-coordinates of childhood points are able to distinguish the two groups over the entire ROI as well as in regions of low uptake and regions of high uptake.

### Within-group comparisons

Heterogeneous uptake behavior cannot be captured by H24 normalized %ID/g or T:H ratio, which are global measures of uptake. However, within-group comparisons of persistence points and childhood points provide information regarding heterogeneity of uptake between the targeting antibody and the control antibody.

When separating the tumor ROI into high and low regions based on voxel values, the differences between the slopes of the high and low H24 normalized %ID/g calculations are not significant in both Group 1 and Group 2 (Table 4a). In contrast, separating the persistence points and childhood points into high and low subsets based on the value of their corresponding maxima demonstrates that both the time series persistence diagrams and time series childhood diagrams can capture heterogeneous uptake behavior. In Group 2 the difference between the slopes of the H24 normalized high *y*-coordinates and the H24 normalized low *y*-coordinates is statistically significant, while in Group 1 the difference between these slopes is not significant. This holds for both the time series persistence diagrams and time series childhood diagrams (Table 4b).

We speculate that the significant difference between the high and low regions in Group 2 indicates that there exists heterogenous uptake behavior in Group 2, which is not present in Group 1. Heterogenous behavior is present in uptake of the control antibody since the EPR effect is more prominent in regions of high vascularity (Greish 2010; Heneweer et al. 2010; Srinivasan and Mane 2014). Regions of high uptake correspond to regions of abnormal vascularity, and thus there is more antibody accumulation in regions of high uptake than in regions of low uptake in Group 2. While the EPR effect causes antibody accumulation in high regions for the targeting antibody as well, the blood clearance rate of the targeting antibody is greater than that of the control antibody, and targeting antibodies also bind to receptors and are internalized by tumor cells in high regions. Therefore, since we see homogenous behavior in targeting antibody uptake behavior it is harder to differentiate the high regions where the antibodies accumulate but then are both cleared from the blood at a fast rate and internalized by tumor cells. %ID/g is a global measure of the tumor and therefore it cannot capture the heterogenous uptake behavior that we are seeing with persistence and childhood data.

Considering heart normalization, T:H ratio, heart normalized *x*-coordinates of the persistence points and childhood points, and the heart normalized *y*-coordinates of the persistence points and childhood points all capture statistically significant differences between uptake rates in the high and low tumor regions in both Group 1 and Group 2 (Table 4). After controlling for clearance we see heterogenous behavior in Group 1 with the H24 normalized *y*-coordinates, and this behavior was not present in Group 1 with respect to the H24 normalized persistence points. This indicates that in regions of high uptake the targeting antibody is accumulating and binding to cell receptors at a faster rate than in regions of low uptake. With the H24 normalization the high clearance rates of the targeting antibody mask the effects of the targeting antibody’s heterogenous binding and accumulation behavior.

The differences between the high and low regions of the tumors show that T:H ratio, the heart normalized time series persistence diagrams, and the heart normalized time series childhood diagrams all capture heterogeneous binding and accumulation behavior in tumor ROI. This heterogenous behavior is present with both the targeting antibody and the control antibody. That is, these methods all satisfy the second of the properties. In short, with respect to heart normalization, all of the methods presented detect heterogeneity of uptake between high and low segmented regions within a group. However, the H24 normalized *y*-coordinates are uniquely able to differentiate tumor heterogeneity between the groups.

## Conclusion

In this paper we present a method that employs computational topology to quantify heterogenous uptake behavior across a time series of SPECT images. This method involves persistence diagram and childhood diagram representations of local behavior in tumor ROI. We apply these methods to differentiate antibody uptake behavior in a group of mice injected with a targeting antibody from a group of mice injected with a control antibody.

Overall, persistence surpasses standard aggregate measures in their ability to (i) directly distinguish between targeting and control antibody uptake rates, (ii) directly distinguish between in-group high and low region uptake rates, and (iii) distinguish between targeting and control antibodies indirectly by displaying different patterns of statistical significance in the in-group high and low region uptake rates. Further, the childhood diagrams provide more local information than the persistence diagrams and therefore the childhood diagrams more effectively distinguish between targeting and control antibody uptake rates while also capturing tumor heterogeneity. Notably, the childhood points alone distinguish the overall uptake behavior between the two groups and the local uptake behavior between the two groups using either normalization.

Our method of applying a topological analysis to targeted In-111 uptake in SPECT images of murine tumors can provide quantifiable information regarding local antibody binding, clearance, and accumulation rates of targeting antibody and control antibody. The results of this analysis show that our method can directly distinguish between targeting antibody and control antibody uptake rates, differentiate between in-group high and low region uptake rates, and distinguish between targeting and control antibodies indirectly by displaying different patterns of statistical significance in the in-group high and low region uptake rates.

The study under consideration had a particular design: the mice were injected with the same tumor cell line and the groups were distinguished by being injected with different In-111 labeled antibodies. There are, however, many possible study designs for assessing different features of tumor cell lines and antibodies. These are quite specific. For example, a different choice of targeting antibody would yield different clearance and binding rates. Another possibility would be to study the uptake of different cancer cell lines also for pre-clinical use. In this case there might be a single targeting antibody so that the analysis might reveal information about differential binding to the different cell lines. The methods we have developed here would be applicable to these and other situations. The statistical analysis also extends naturally to more than two study groups. When applied to other study designs, analyses based on persistence diagrams and childhood diagrams would be expected to yield local information about heterogeneous uptake behavior that is not detectable by the standard aggregate methods.

## Appendix

### Standard analyses

See Figs. 8, 9 and 10.

### Kruskal–Wallis results

See Figs. 11 and 12.

### Linear trend comparisons

See Figs. 13, 14 and 15.

### Tables

In the following tables, *p* values less than $10^{-4}$ are reported as 0 and *p* values indicating statistical significance are in bold.

See Tables 1, 2, 3 and 4

**Table 1:** Kruskal–Wallis results

Data	*p* value	
(a) Standard metrics		
G1 %ID/g	**0.031**	
G2 %ID/g	0.551	
G1 T:H ratio	**0.039**	
G2 T:H ratio	0.078	

Data	Persistence	Childhood
	*p* value	*p* value
(b) Persistence points and childhood points		
G1 H24 norm x-coords	0.740	0.746
G2 H24 norm x-coords	0.665	0.767
G1 H24 norm y-coords	**0.000**	**0.000**
G2 H24 norm y-coords	**0.000**	**0.000**
G1 heart norm x-coords	**0.000**	**0.000**
G2 heart norm x-coords	**0.005**	**0.002**
G1 heart norm y-coords	**0.000**	**0.000**
G2 heart norm y-coords	**0.000**	**0.000**

**Table 2:** Slopes of best fit lines

Data	Slope all	Slope high	Slope low
(a) Standard metrics: slopes of all, high, and low voxels			
G1 %ID/g	− 0.043	− 0.078	− 0.007
G2 %ID/g	0.031	0.111	0.007
G1 T:H ratio	0.010	0.035	0.006
G2 T:H ratio	0.009	0.027	0.003
(b) Persistence points: slopes of all, high, and low points			
G1 H24 norm x-coords	$-\,3.25 \times 10^{-4}$	$5.216 \times 10^{-4}$	$-3.739 \times 10^{-4}$
G2 H24 norm x-coords	0.002	0.020	$5.719 \times 10^{-4}$
G1 H24 norm y-coords	− 0.013	− 0.001	− 0.013
G2 H24 norm y-coords	0.011	0.034	0.010
G1 heart norm x-coords	0.005	0.047	0.003
G2 heart norm x-coords	0.002	0.021	0.001
G1 heart norm y-coords	0.026	0.046	0.025
G2 heart norm y-coords	0.017	0.033	0.015
(c) Childhood points: slopes of all, high, and low points			
G1 H24 norm x-coords	$9.284 \times 10^{-4}$	0.017	$1.420 \times 10^{-4}$
G2 H24 norm x-coords	0.001	0.012	$8.040 \times 10^{-4}$
G1 H24 norm y-coords	− 0.013	− 0.009	− 0.013
G2 H24 norm y-coords	0.011	0.038	0.009
G1 heart norm x-coords	0.004	0.035	0.002
G2 heart norm x-coords	0.001	0.008	0.001
G1 heart norm y-coords	0.027	0.052	0.025
G2 heart norm y-coords	0.017	0.039	0.016

**Table 3:** Group 1 versus Group 2 linear trend comparison *p* values

Data	All	High	Low			
(a) Standard metrics						
%ID/g	**0.019**	**0.022**	0.211			
T:H ratio	0.687	0.530	0.201			

Data	Persistence points			Childhood points		
	All	High	Low	All	High	Low
(b) Persistence points and childhood points						
H24 norm y-coords	**0.000**	0.062	**0.000**	**0.000**	**0.016**	**0.000**
Heart norm x-coords	0.222	0.273	**0.036**	**0.006**	**0.008**	**0.008**
Heart norm y-coords	**0.000**	0.224	**0.000**	**0.000**	0.274	**0.000**

**Table 4:** Within Group high versus low linear trend comparison *p* values

Data	Group 1	Group 2		
(a) Standard metrics				
%ID/g	0.065	0.139		
T:H ratio	**0.006**	**0.004**		

Data	Persistence points		Childhood points	
	Group 1	Group 2	Group 1	Group 2
(b) Persistence points and childhood points				
24 norm y-coords	0.177	**0.042**	0.652	**0.018**
Heart norm x-coords	**0.000**	**0.000**	**0.000**	**0.000**
Heart norm y-coords	**0.030**	**0.001**	**0.006**	**0.000**

## Footnotes

1 A voxel is the three-dimensional analog of a pixel.
